# A Rare Case of a Spontaneously Ruptured Hepatocellular Adenoma in the Third Trimester of Pregnancy

**DOI:** 10.7759/cureus.72447

**Published:** 2024-10-26

**Authors:** Ismini Kountouri, Eftychia Kokkali, Amyntas Giotas, Ioannis Katsarelas, Periklis Dimasis

**Affiliations:** 1 Department of General Surgery, General Hospital of Katerini, Katerini, GRC; 2 Department of Radiology, General Hospital of Katerini, Katerini, GRC; 3 Department of Obstetrics and Gynecology, General Hospital of Katerini, Katerini, GRC; 4 Department of Surgery, General Hospital of Katerini, Katerini, GRC

**Keywords:** emergency cesarean section, hepatocellular adenoma, liver tumor in pregnancy, maternal hemodynamic instability, pregnancy complications, ruptured liver tumor

## Abstract

This case report details the management of a 29-year-old primigravida who presented at 35 weeks of gestation with abdominal pain and vomiting. The patient exhibited tachycardia and fetal bradycardia, with laboratory findings indicating severe anemia and elevated liver enzymes. An emergency cesarean section was performed due to hemodynamic instability, during which a ruptured hepatocellular adenoma (HCA) was discovered, necessitating an atypical left hepatectomy. Despite successful maternal recovery, the neonate succumbed to multiple organ failure. Hepatocellular adenomas (HCA), also known as hepatic adenomas, are rare benign epithelial liver tumors that predominantly occur in women during their reproductive years and have been strongly associated with the intake of oral contraceptives. This case highlights the rare occurrence of HCA rupture during pregnancy, emphasizing the importance of prompt diagnosis and intervention to prevent life-threatening complications. Additionally, it underscores the need for careful monitoring and potential preemptive intervention in pregnant women with large HCAs due to the elevated risk of rupture.

## Introduction

Hepatocellular adenomas (HCA), also known as hepatic adenomas, are rare benign epithelial liver tumors [1]. They predominantly occur in women during their reproductive years and have been strongly associated with the intake of oral contraceptives (OC) [1-3]. The prevalence of these tumors is not exactly known with studies showing an estimated incidence of 1-1.3 per 1,000,000 in women who have never used oral contraceptives (OC), compared to 30-40 per 1,000,000 in long-term users [2]. Other identified risk factors include steroid abuse, Fanconi anemia, aplastic anemia, metabolic syndrome, and glycogen storage disease (GSD) [1]. Clinical symptoms associated with HCAs may include right upper quadrant abdominal pain or discomfort due to intratumoral bleeding, elevated liver enzymes, and signs of life-threatening hemorrhage into the peritoneal cavity, such as shock [2]. This case highlights the rare occurrence of HCA rupture during pregnancy, emphasizing the importance of prompt diagnosis and intervention to prevent life-threatening complications. Additionally, it underscores the need for careful monitoring and potential preemptive intervention in pregnant women with large HCAs due to the elevated risk of rupture.

## Case presentation

A 29-year-old primigravida presented to the emergency department of the General Hospital of Katerini, Greece, with complaints of abdominal pain and vomiting. The patient was 35 weeks pregnant, had no prior history of liver disease, and reported no recent trauma. On clinical examination, she was tachycardic with a heart rate of 135 beats per minute, and the fetal heart rate was notably low at 70 beats per minute. Laboratory results revealed a hemoglobin level of 6.8 g/dL, hematocrit at 21.0%, and a white blood cell count of 16.2 x 10^3^/μL. The complete blood count upon admission is shown in Table 1. The biochemical profile indicated elevated levels of serum glutamic-pyruvic transaminase (SGPT) at 127.1 U/L and serum glutamic-oxaloacetic transaminase (SGOT) at 188.6 U/L. A transabdominal ultrasound did not show any abnormalities in the fetus, but a significant amount of free intra-abdominal fluid was detected. Given the patient’s deteriorating hemodynamic status and the extremely low fetal heart rate, an emergency cesarean section was performed.

**Table 1:** Complete blood count of the patient upon admission

Complete Blood Count	
Red Blood cell (RBC) count	2.23 × 10^6^/μL ( Reference range: 3.8 - 5.8× 10^6^/μL )
White blood cell (WBC) count	16.2 × 10^3^/μL ( Reference range: 4 - 11 × 10^3^/μL )
Hemoglobin (Hb)	6.8 g/dL (Reference range: 12 -16 g/dL )
Hematocrit (HCT)	21.0% ( Reference range: 36 - 47 % )
Patelet (PLT) count	83 × 10^3^/μL ( Reference range: 150 - 400 × 10^3^/μL )

**Figure 1:**
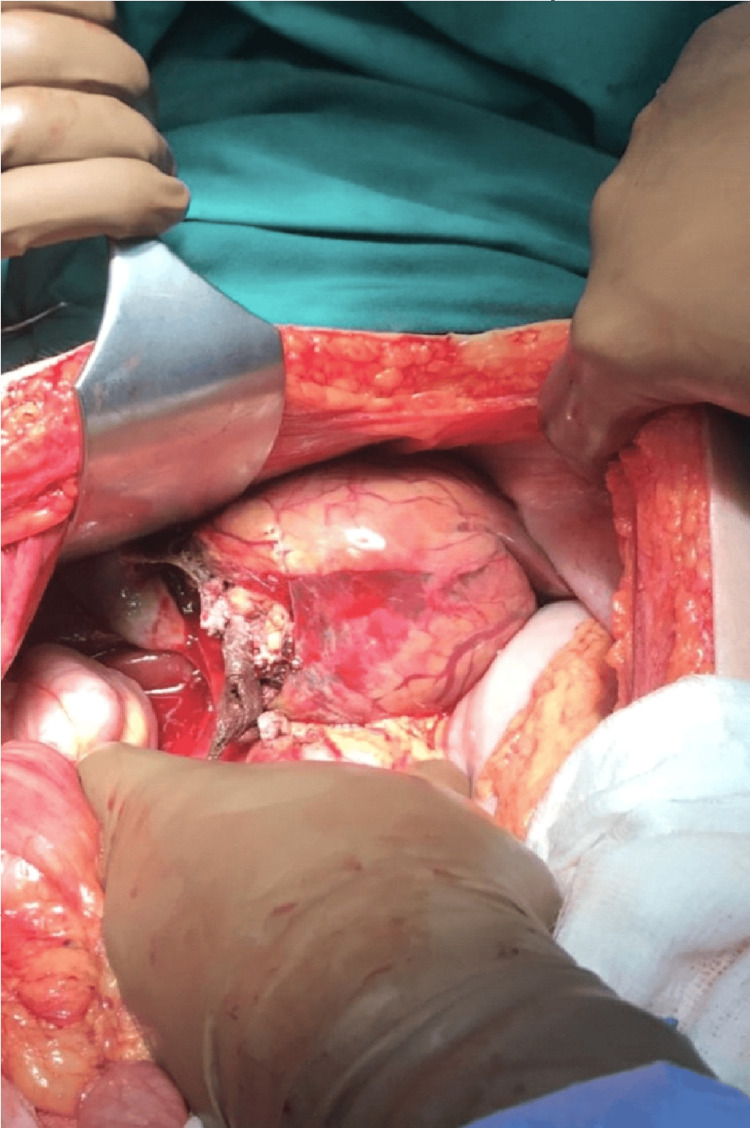
Intraoperative image showing the large hepatic tumor and the rupture site.

**Figure 2:**
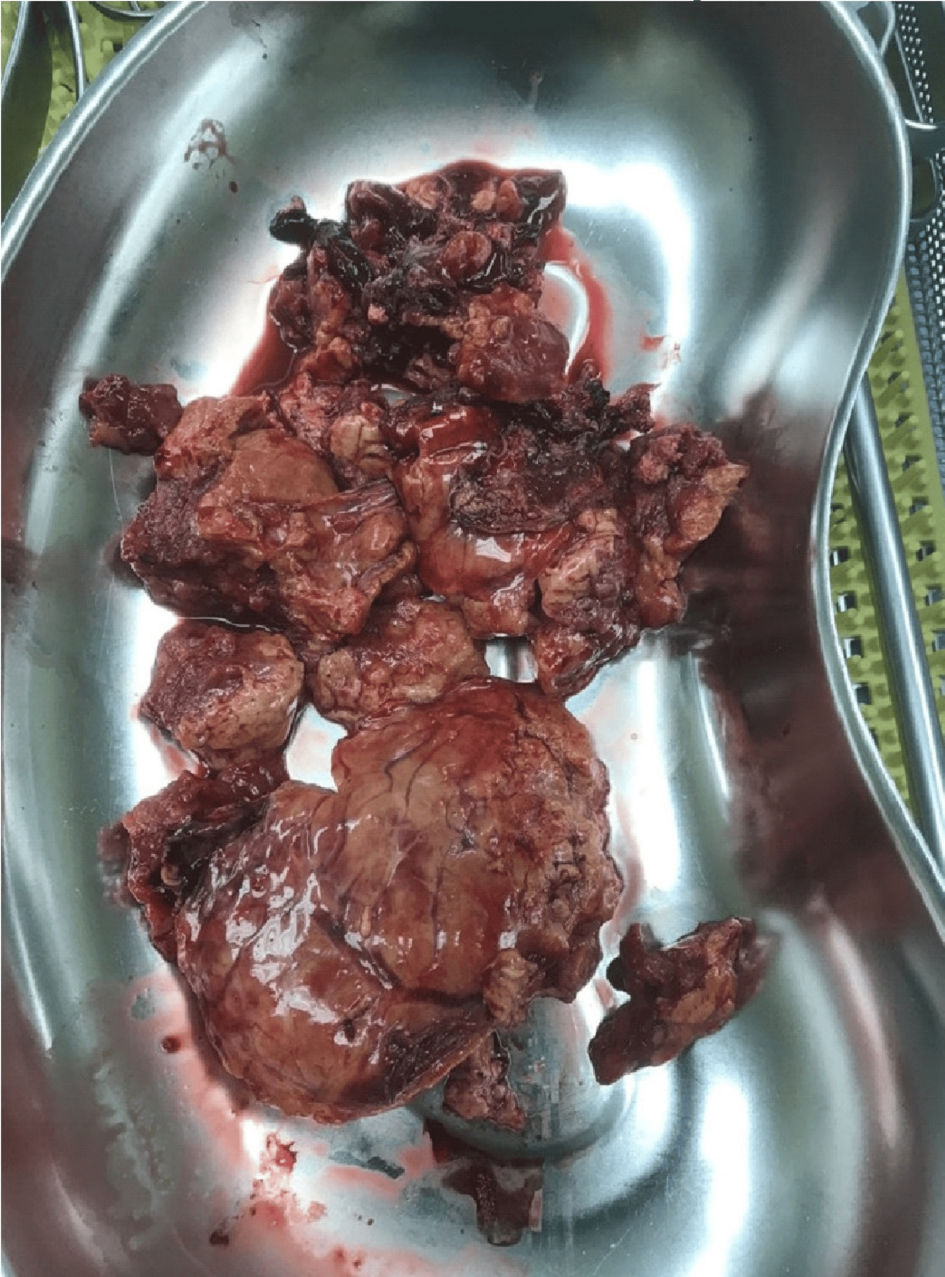
The hepatectomy specimen submitted for histopathological examination.

During the laparotomy through a Pfannenstiel incision, the infant was delivered alive, but the uterus failed to contract despite the maximum oxytocin and ergonovin administration, leading to uncontrolled bleeding. Consequently, a hysterectomy was performed. Following the hysterectomy, persistent intraperitoneal bleeding unrelated to the procedure necessitated the involvement of the general surgery team. Through a Pfannenstiel incision, a liver mass was palpated, prompting the decision to perform an upper midline incision for better evaluation and control of the liver hemorrhage. The left lobe of the liver was found to contain a large, ruptured hepatic tumor with active bleeding (Figure 1). Initial efforts to control the hemorrhage with pressure and cautery were unsuccessful, leading to an atypical left hepatectomy using a gastrointestinal anastomosis (GIA) stapler (Figure 2).

**Figure 3:**
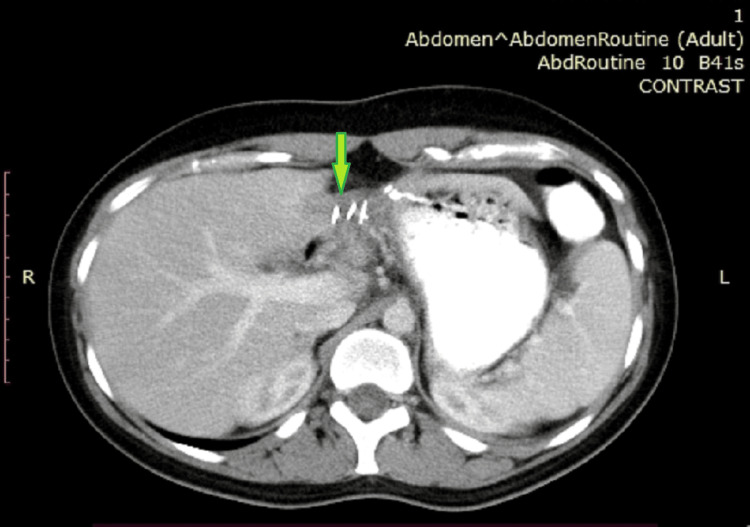
Postoperative CT scan showing no detectable liver pathology. The hepatectomy site is visible on the left and marked by a green arrow. The clips from the GIA stapler are also visible.

Postoperatively, the patient was transferred to the intensive care unit (ICU), where she gradually stabilized and was successfully extubated. She was later moved to the surgical ward and had an uneventful recovery. Histopathological examination confirmed the presence of a large HCA measuring 7.9 cm x 6.2 cm x 2.6 cm. No abnormalities were found in the histopathology of the uterus. The macroscopic examination showed the presence of numerous, irregular, soft, yellow-gray or brown-gray tissue pieces with a total weight of 188 grams. Microscopically, the neoplastic lesion had histopathological features of an HCA. The cells of the lesion were arranged in trabeculae a few cells thick and characterized by granular eosinophils or clear cytoplasm. Their nuclei were smooth and sub-round with prominent nucleoli and localized bi-nucleate forms. Arranged atrial capillaries were observed between the cells of the lesion, as well as scattered arterioles, without the presence of bile vessels. Mitosis, particularly atypia, pseudo-adenoid formations, necrosis, steatosis, ischemic lesions, or hemorrhages were not identified. Neoplastic cells were negative for β-catenin, while most atrial capillaries among the neoplastic cells were CD34 positive. A follow-up CT scan one year after the initial surgery revealed no liver pathology or relapse (Figure 3). Unfortunately, despite medical intervention, the newborn was admitted to the neonatal intensive care unit and succumbed to multiple organ failure three days later.

## Discussion

HCAs are often asymptomatic and are typically discovered incidentally during abdominal ultrasonography conducted for unrelated reasons [2]. Prompt diagnosis is crucial for women of reproductive age due to the potential for rapid tumor growth and rupture [2]. An association between sex hormones, notably oral contraceptive pills and anabolic steroids, and HCA development has been reported in the literature. Hence, these tumors predominantly occur in women during their reproductive years [1-3]. In addition to the risk of rupture, HCAs carry a reported risk of malignant transformation, occurring at a rate of approximately 4.2% [4]. Symptoms associated with the presence of HCAs are a right upper quadrant abdominal pain or discomfort due to intratumoral bleeding, elevated liver enzymes, and signs of life-threatening hemorrhage into the peritoneal cavity, such as shock [2].

For male patients, tumor excision is recommended regardless of tumor size. In female patients, excision is generally advised for tumors larger than 5 cm because of the risk of hormone-induced growth and spontaneous rupture due to increased levels of steroid hormones during pregnancy [1-2]. Additionally, women with HCAs should be counseled to discontinue the use of oral contraceptives and other hormone-based medications, with observation being the initial approach to treatment [2]. Surgical resection is typically indicated if the tumor exceeds 5 cm after six months of observation without OC use, fails to regress adequately after OC discontinuation, rebleeds, or if there is any diagnostic uncertainty [5-10].

Because of the high risk of complications during pregnancy, women with large tumors should be advised to undergo intervention (surgery, radiofrequency angioembolization (RFA), or embolization) before becoming pregnant [11]. In cases of HCA during pregnancy, a "watch and wait" approach is generally accepted in the literature [12]. A prospective study by Gaspersz et al. in 2019 examined the biological behavior of small HCAs during pregnancy and suggested that while HCAs smaller than 5 cm pose minimal risk to the mother and no risk to the child, close monitoring with ultrasound is recommended for tumors that grow in size, allowing for intervention if necessary [13].

When intervention is required, such as in cases of rupture or bleeding [14], minimally invasive techniques are generally recommended. The Society of American Gastrointestinal and Endoscopic Surgeons (SAGES) issued guidelines in 2011 for the diagnosis, treatment, and use of laparoscopy for surgical problems during pregnancy, suggesting that a laparoscopic approach is safer for both the mother and the fetus [11,12]. A systematic review by Wilson et al. concluded that RFA and formal resection in cases of HCA hemorrhage during pregnancy are safe, yielding good clinical outcomes [15]. RFA is recommended as an alternative treatment for women with HCA and is considered relatively safe in experienced hands, with low mortality and morbidity [12]. However, a limitation of RFA is the lack of tissue available for pathological examination after ablation, and the risk of radiation exposure to the fetus, particularly before 26 weeks of gestation, making it less desirable during early pregnancy [11].

Other cases similar to ours have been reported in the recent literature. In 2018, Gryspeerdt et al. reported on a case of laparoscopic liver resection for hemorrhagic HCA in a pregnant patient who, at 18 weeks of gestation, underwent a semi-elective laparoscopic left lateral sectionectomy and later delivered a healthy neonate at 40 weeks of gestation [16]. In 2018, Bernède et al. also reported on a spontaneous hemoperitoneum in pregnancy caused by an HCA, that was accompanied by fetal death and was managed only by perihepatic gauze packing [17]. In 2021, Olesen et al. reported on a patient with an HCA as the cause of liver rupture and intrauterine fetal death. This patient was successfully managed with transarterial embolization and cesarean section, followed by laparoscopic liver resection four weeks later [18]. Laparoscopic resection is generally recommended for ruptured HCAs during pregnancy, following initial stabilization of the patient [11,12,15].

## Conclusions

In cases like ours, where the HCA was unknown prior to surgery and was first diagnosed during laparotomy, prompt decision-making is essential, with a damage control approach being the method of choice. Given the nonspecific presentation and the limitations of diagnostic imaging during pregnancy, these cases can often be underdiagnosed, leading to potentially life-threatening complications for both the mother and fetus. We recommend that such patients be treated with the best available option, which in most cases includes delivery of the fetus and primary liver resection and tumor excision via laparotomy. A ruptured HCA is a surgical emergency, and this case report aims to raise awareness among both surgeons and obstetric specialists to remain vigilant in managing these cases.
